# Single neurons in the human substantia nigra encode social learning signals

**DOI:** 10.64898/2026.09.18.752742

**Published:** 2026-09-20

**Authors:** Arianna Neal Davis, Ofer Perl, Brian H Kopell, Matthew Heflin, Qi Xiu Fu, Salman E Qasim, Zarghona Imtiaz, Ayaka Kato, Soojung Na, Navid Mikail, Helen S Mayberg, Read Montague, Ignacio Saez, Xiaosi Gu

## Abstract

Humans are remarkable at adapting to changing norms, yet we know little about how the human brain encodes these social signals at the single neuron level. Here, we recorded the activity of single neurons in the substantia nigra and globus pallidus in neurosurgical participants as they played an iterative ultimatum game versus human and computer avatars. Using computational modeling of behavior, we found that putative dopaminergic units in the substantia nigra tracked the valence of norm prediction errors, with greater encoding during gameplay with human versus computer avatars. Analogous analyses in the globus pallidus, a nucleus adjacent to the substantia nigra, found no significant effect. Our results identify a novel role for the substantia nigra in encoding social learning signals.

## Full Text Availability

The license terms selected by the author(s) for this preprint version do not permit archiving in PMC. The full text is available from the preprint server.

